# How to Interpret an Investigator’s Brochure for Meaningful Risk Assessment: Results of an AGAH Discussion Forum

**DOI:** 10.1007/s43441-021-00257-0

**Published:** 2021-02-03

**Authors:** Jens Rengelshausen, Kerstin Breithaupt-Groegler, Frank Donath, Katharina Erb-Zohar, Tim Hardman, Gerd Mikus, Stephanie Plassmann, Georg Wensing, Hildegard Sourgens

**Affiliations:** 1 Association for Applied Human Pharmacology (AGAH e.V.), Goernestrasse 30, 20249 Hamburg, Germany; 2 Association for Human Pharmacology in the Pharmaceutical Industry (AHPPI), London, UK; 3 Grünenthal GmbH, Clinical Science, Zieglerstr. 6, 52078 Aachen, Germany; 4 -kbr- Clinical Pharmacology Services, 60431 Frankfurt am Main, Germany; 5 SocraTec Research and Development GmbH, 99084 Erfurt, Germany; 6 clinphase®, 63679 Schotten, Germany; 7 Niche Science & Technology, Unit 26, Falstaff House, Bardolph Road, Richmond, TW9 2LH UK; 8 Department of Clinical Pharmacology and Pharmacoepidemiology, University of Heidelberg, 69120 Heidelberg, Germany; 9 PreClinical Safety (PCS) Consultants Ltd, Nauenstrasse 49, 4052 Basel, Switzerland; 10 Bayer AG, Clinical Pharmacology, 42096 Wuppertal, Germany; 11 Sourgens Consulting, 80797 Munich, Germany

**Keywords:** Investigator’s brochure, Investigational medicinal product, Clinical trial, First-in-human, Risk assessment

## Abstract

**Purpose:**

A discussion forum was hosted by the Association for Applied Human Pharmacology (AGAH e.V.) to critically debate how to interpret and optimise the Investigator’s Brochure (IB) for meaningful risk assessment of early clinical trials.

**Materials and Methods:**

Four topics were specifically discussed: deficiencies/uncertainties in IBs, guidance for the investigator, reference safety information, and potential risks for human subjects associated with inadequate non-clinical safety assessment in the IB. In each case, 43 participants took part in a real-time online survey with pre-defined questions to capture the audience’s opinion.

**Results:**

The ‘Summary of Data and Guidance for the Investigator’ was considered as the section of the IB with the highest need for improvement with emphasis on readability, comprehensibility, timeliness of data, and appropriateness for risk assessment. It was suggested that the IB should at least be signed by the sponsor’s scientist responsible for the content on pharmacology and toxicology. It was agreed that sponsors should consider thoroughly whether changes to an IB constitute a substantial amendment, and that the IB should include a section on the change history. Non-clinical pharmacology studies with negative outcomes should be reported in the IB in order to avoid assessment bias. The reference safety information for expectedness assessment of suspected serious adverse reactions should be provided as a stand-alone section of the IB.

**Conclusion:**

The overall consensus was that an optimised presentation of data will ensure the best possible understanding of a compound’s characteristics and an optimal benefit-risk assessment which will safeguard the participants in clinical trials.

## Introduction

The Investigator’s Brochure (IB) is a multifunctional regulatory document essential for the conduct of clinical trials that summarises the physical, chemical, pharmaceutical, pharmacological, and toxicological characteristics of an investigational medicinal product (IMP) as well as any clinical experience. The ICH E6 (R2) Guideline for Good Clinical Practice recommends a standardised content structure for the IB which includes a final summary of data and guidance for the investigator (Table 1) [1]. One key function of the IB is to provide investigators with data about the IMP that informs them in their conduct of a clinical trial, providing information that can facilitate the understanding of potential safety concerns and appropriate precautions to minimise the risk of exposure in healthy subjects and patients receiving the IMP.

**Table 1.** Table of Contents of the IB as Exemplified in ICH E6 (R2) [1].

Section	Content
–	Confidentiality statement (optional)
–	Signature page (optional)
1	Table of contents
2	Summary
3	Introduction
4	Physical, chemical, and pharmaceutical properties and formulation
5	Non-clinical studies
5.1	Non-clinical pharmacology
5.2	Pharmacokinetics and product metabolism in animals
5.3	Toxicology
6	Effects in humans
6.1	Pharmacokinetics and product metabolism in humans
6.2	Safety and efficacy
6.3	Marketing experience
7	Summary of data and guidance for the investigator

Safety remains the primary concern of those conducting early phase clinical trials. However, during the early stages of development, clinical experience with the IMP is either lacking (first-in-human studies; FIH) or sparse, leaving assessment of risk dependent on non-clinical pharmacology, safety and toxicology data when transitioning from in vitro investigations and animal studies to humans. While more than 3000 FIH trials have been conducted in the European Union (EU) [2] over the last 14 years, there have been two incidents where exposure to IMP has resulted in tragic consequences: the serious systemic inflammatory responses (cytokine storm) following the first administration of TGN1412 in 2006 [3] and the serious neurologic adverse effects after multiple high-dose administrations of BIA 10-2474 in 2016, leading to the death of one healthy subject [4]. In considering circumstances behind these events the European Medicines Agency (EMA) issued a revised guideline on strategies to identify and mitigate risks to subjects involved in first-in-human and early clinical trials [5]. The guideline requires sponsors to describe the degree of uncertainty around the predicted effects of the IMP which can then be used to determine corresponding risk mitigation strategies. Those involved in the design, approval and conduct of clinical trials are then required to base any decisions they make before and during a trial on rigorous assessment of all the available data—with the Investigator’s Brochure being the pivotal document where these data can be found. Consequently, the clear presentation of IMP-related data within the IB with its interpretation of potential risks and appropriate risk mitigation measures is of utmost importance for the safe and successful conduct of clinical trials.

## Materials and Methods

A discussion forum to critically debate on how to optimise the presentation of data in the Investigator’s Brochures and derive a meaningful assessment of risk in early clinical trials was hosted by the German non-for profit Association for Applied Human Pharmacology (AGAH e.V.) in Bonn, Germany on November 11, 2019. Participants comprised expert stakeholders from pharmaceutical industry, clinical contract research organisations (CROs), scientific consultancy, academia, ethics committees and the German competent regulatory authority, the Federal Institute for Drugs and Medical Devices (BfArM), (for conference details, contributions, and presentations see https://www.agah.eu/event/agah-diskussionsforum-2019/).

After a keynote lecture entitled ‘The IB—key information to support early clinical trials’, the following four topics were first introduced by brief presentations and then discussed with the audience:

- Deficiencies/uncertainties in IBs—quality of IB sections 1 to 6
- Guidance for the investigator—quality of IB section 7
- Reference safety information in the IB
- Potential risks for human subjects associated with inadequate non-clinical safety assessment in the IB

At the end of each discussion session, the participants were asked to voluntarily take part in an online survey using the online slido tool (https://www.sli.do/) on their own mobile devices and answer a series of specific questions either by choosing from a panel of multiple pre-specified answers or by giving a rating from a score between 1 = very bad and 5 = very good, as applicable. The intention of the online survey was to capture the opinions and experience of the participants in a structured way. Since this survey was not regarded as medical research or a clinical study, an explicit written informed consent was not obtained from the participants. Responses were compiled electronically. The survey responses underwent real-time analysis by the slido tool to provide descriptive statistical results which comprised percentage of participants who have given a specific answer and mean values for rating scores where applicable.

Forty-three participants of the discussion forum took part in the online survey. Six of them (14%) came from the pharmaceutical industry, 20 (47%) from CROs, seven (16%) from a regulatory authority, six (14%) from universities, one (2%) from an ethics committee, and three (7%) were scientific consultants. The majority of participants were based in Germany (38; 88%), three (7%) were from the Netherlands and two (5%) from Switzerland.

The results presented are reflecting the discussion of the experts at the forum based on their opinions and experience. The online surveys add to this approach, but it is not intended to fulfil the characteristics of systematic research or a clinical study.

## Results and Discussion

### Translational Integration of Non-clinical Data from the Investigator’s Brochure

The keynote lecture addressed better ways for displaying and interpreting data within IBs and focused on the recent development of a tool that can be used to integrate and display specific data [6]. The tool consists of a database in which relevant results from non-clinical studies on the IMP are entered. The tool enables data to be sorted in tabulated rows by increasing maximum systemic drug concentrations (*C*_max_) or by increasing estimated human equivalent doses. Missing data on *C*_max_ may need to be interpolated from population pharmacokinetic models or from other studies within the same species. Results from pharmacology, safety, and toxicology studies can be differentiated by colour (e.g. blank = no data, green = pharmacologically or therapeutically desirable, yellow-orange = increasingly severe reversible adverse events, red = irreversible toxicity and death). Adding an anticipated human effective maximum concentration or dose or even data from first clinical trials allows the sponsor and the investigator to get an overview on all non-clinical data for the IMP and to obtain an overview of whether the intended human doses are expected to be safe for administration in humans. Several real-world examples illustrated the practicability of the tool which is freely available for use (www.ib-derisk.org) [6].

The emphasis of this approach is on acute, potentially adversely exaggerated pharmacological effects correlating with *C*_max_. Dose-limiting acute adverse responses to substances of certain classes, such as compounds causing pharmacologically mediated functional central nervous system (CNS) effects, may directly correlate with maximal systemic plasma/serum concentrations and are a typical function of the pharmacological on-target activity [7]. However, for most drug classes, including CNS-active drugs, the full characterisation of the safety profile requires repeated dosing and exposure data in terms of area under the concentration–time curve (AUC) which are often more predictive of how effects will translate from one species to another, with the exception of acute functional effects [8].

### Deficiencies/Uncertainties in IBs: Quality of IB Sections 1 to 6

The German Medicinal Products Act requires that ‘each investigator has been informed by a scientist responsible for the pharmacological-toxicological test about the findings of the test and the foreseeable risks involved in the clinical trial’ [9]. Although this information is provided in the IB, this prerequisite to conducting clinical trials with the IMP has the inherent implication that an identifiable scientist takes responsibility for the acceptability of the risk to subjects by providing the signed approval in the IB. Deficiencies observed by the BfArM refer to the use of different IB versions in parallel and to IB changes considered as non-substantial by the sponsor although they had to be regarded as substantial according to the assessment by BfArM.

The availability of new non-clinical and/or clinical data is usually considered substantial as they could have an impact on safety of subjects in clinical trials and need to be scientifically reviewed by the national competent authority, whereas non-substantial amendments are processed administratively only. The sponsor must assess any new information to evaluate its potential impact on the established risk/benefit profile. The BfArM requires the sponsor to provide this assessment for the IMP and the ongoing trials, justifying why either the risk/benefit assessment remains unchanged or why existing measures are considered adequate to control for any newly identified risks or previously established risks with an altered appraisal (e.g. lower NOAEL with prolonged treatment but same principal target organ).

It was general consensus that similar to clinical trial protocols, the IB should include a last section on the amendment change history. Overall, it is the task of the sponsor to constantly evaluate the risk/benefit of an ongoing clinical trial, and the competent authority must be able to scrutinise this assessment objectively.

The findings of the online survey asking participants about the overall content quality of the IB they have previously used for early development clinical trials revealed a mean score of 3.6 (where a score of 1 = very bad and 5 = very good). The majority of participants (55%) gave a rating of 4. More detailed participant questioning revealed mean scores of 3.1 for readability, 3.3 for comprehensibility, 3.3 for appropriateness for risk assessment, 3.6 for timeliness, 3.3 for quality of presented non-clinical information, and 3.7 for quality of presented clinical information.

### Guidance for the Investigator: Quality of IB Section 7

Important requirements for this section of the IB from the viewpoint of the investigator are completeness and correctness of data as well as clarity, comprehensibility, and relevance of the information provided [10]. It is generally agreed that this is best achieved through the logical presentation of data, indication of important missing data, and comprehensive conclusions containing guidance for dosing, warning messages on adverse reaction that might be expected with the IMP, and recommendations on appropriate measures to deal with IMP-related medical emergencies.

From the point of view of the ethics committee, non-clinical studies on pharmacology should include elements essential for ensuring validity such as randomisation, sample size calculation, and blinded outcome assessments [11]. Non-clinical pharmacology studies to establish the proof-of-principle for a given investigational medicinal product (IMP) are ideally undertaken in various disease models since many of them face limitations regarding their predictive value for the human condition, and a combination of studies provides a more meaningful basis to assess the relevance of the data. Typically, some studies may not show evidence of efficacy whereas others are positive. However, all pharmacology studies including those with negative outcomes should be reported in order to avoid assessment bias and to allow investigators to appraise the strength of the supporting preclinical data systematically which should be reflected in the guidance for the investigator [11].

### Reference Safety Information in the IB

According to the ‘detailed guidance on the collection, verification and presentation of adverse event/reaction reports arising from clinical trials on medicinal products for human use’ issued by the European Commission [12] and according to ‘regulation (EU) no 536/2014 of the European Parliament and of the Council on clinical trials on medicinal products for human use’ [13], the IB shall include a clearly identifiable section called the ‘Reference Safety Information’ (RSI). The RSI shall contain information on the IMP, how to determine whether adverse reactions should be considered as expected adverse reactions, and on the expected frequency and nature of such adverse reactions [13]. The sponsor’s informed opinion on the expectedness of an adverse reaction must be provided from the perspective of previously observed events rather than the basis of what might be anticipated from the pharmacological properties of the IMP [12].

More specific guidance was recently issued in the form of a Questions & Answers publication by the Clinical Trial Facilitation Group (CTFG) [14]. It was highlighted how the RSI should be used by the sponsor for the assessment of the expectedness of all suspected serious adverse reactions (SARs) occurring in clinical trials in order to assess the need for expedited safety reporting [15]. It was determined that the content of the RSI should include a clear list of ‘expected SARs’, i.e. SARs that could be expected following exposure to the IMP. The list should be restricted to ‘suspected’ SARs that had previously been observed and where, after a thorough assessment by the sponsor, reasonable evidence of a causal relationship between the event and the IMP had been established [14]. By implication, each ‘expected SAR’ should already have been reported as a ‘suspected’ SAR more than once. Where SARs turned out to be fatal and life-threatening, they would usually be considered to be unexpected even if the fatal or life-threatening SARs had been reported previously [14]. In the IB, the sponsor should clearly indicate that the RSI section outlines expected SARs for regulatory reporting purposes and that the information in the RSI section does not present a comprehensive overview of the safety profile of the IMP. Therefore, the RSI should always be provided in a clearly separated and specific section of the IB [13].

It is required that the RSI be presented in a table form where the ‘expected SARs’ are listed by body system organ class using preferred terms as per the latest MedDRA version followed by frequency of incidence calculated on an aggregated level based on previously observed ‘suspected’ SARs. Where necessary, the RSI section of the IB is to be updated annually after the Development Safety Update Report data-lock point by a substantial amendment. All other safety information comprising a detailed overview on the safety profile of the IMP should be provided in the subsection on the ‘Effects in Humans’ and in the ‘Summary of Data and Guidance to the Investigator’ (Section 7) [14, 16].

Following discussion over the RSI, participants indicated through the online survey that most (63%) were familiar with the CTFG Q&A document and that they were used to seeing this information provided as a separate section in some, though not all, of the IBs they had worked with. When debating their involvement in the production of IBs, the majority of participants (61%) indicated that they preferred to locate the RSI as a separate section, Section 8, rather than as a subsection of Section 7. These results are in line with data from a previous web-based survey that was conducted to understand current industry practices in the preparation of safety information for the IB [16].

### Potential Risks for Human Subjects Associated with Inadequate Non-clinical Safety Assessment

The ICH E6 (R2) guideline for Good Clinical Practice requires that results of all relevant non-clinical pharmacology, toxicology, pharmacokinetic, and metabolism studies be provided in a summary form within the IB addressing the methodology and discussing the relevance of the findings to the IMP and the possible unfavourable and unintended effects in humans [1]. It is emphasised that where possible, data should be provided in tabular form to enhance the clarity of its presentation. It was generally agreed that provision of these data in a format that is difficult to interpret would be associated with unwarranted risk in terms of the overall conduct of the clinical trial assuming that critical information could be missed. One example where inadequate presentation might introduce risk would be investigations that had been performed but were not discussed or presented in the IB or cases where the sponsor provides insufficient discussion regarding the relevance of pharmacodynamic models, species differences, off-target effects, safety margins, or findings from non-clinical safety studies. It may also include listing of results in the absence of context and/or a lack of expert interpretation.

Key objectives of the non-clinical safety programme are to identify:

- the initial safe starting dose and subsequent dose escalation schemes in humans;
- potential target organ systems for toxicity including dose dependence of adverse effects, any relation to exposure over the dose range tested (C_max_ and AUC in each species tested including total and free drug including predicted safety margins compared to predicted efficacious exposures in humans), and the assessment of reversibility of adverse effects;
- safety parameters for clinical monitoring [5, 17].

It is the purpose of the IB to describe all these characteristics of the IMP clearly. In particular for early IBs, the results obtained from the non-clinical safety package need to be addressed in appropriate detail and context in order to support the development of a benefit-risk assessment based on the totality of the available evidence. The interpretation of critical findings such as mortality in toxicology studies should be robust and interrogated to establish whether there is any underlying cause for concern, whether it is possible to establish such an opinion with the data available and if so, based on which observations. Key considerations include but are not limited to the nature, type, and severity of the findings across species and studies, their relation to exposures over the dose range tested, the steepness of the dose–response, and predicted safety margins [8]. If any important issues are identified during non-clinical development, it is mandatory to assess critically whether they require clinical development to be put on hold and whether any risks can be mitigated and/or explained. While findings may be deemed to have a negative impact on the risk/benefit profile but the safety of subjects in clinical trials can still be ensured with the overall risk/benefit profile remaining positive, initial measures intended to mitigate potential risks may need to be modified. Any new measures introduced to mitigate risk and address any management needs must be explained clearly and justified. All relevant data should be presented in a transparent manner to convey salient information supporting human risk assessment clearly and concisely.

In debating the production of IBs, it was agreed by the participants that they represent a living document critical to the clinical development of new medicines that are constantly changing as we learn more about an IMP. As such, this places emphasis on producing documents containing the correct information and therefore should be given sufficient time and resources to produce and maintain quality documents, keeping them appropriately updated with new information from all disciplines [10]. Ongoing and interdisciplinary risk assessments must be undertaken regularly and at appropriate junctures to integrate all data, and to identify and address any emerging concerns in a timely manner.

### Possible Improvements to the IB

The final session of the discussion forum involved a group debate on whether it was possible for any parts of the IB to be improved and if so, how that could be delivered. The overall opinion of the participants is summarised in Table 2. The IB section that most of the participants perceived in need for improvement was the summary of data and guidance for the investigator (Section 7), followed by the non-clinical studies (Section 5) and the effects in humans (Section 6). It was generally perceived that this represented a call to the sponsors of clinical trials and the authors of the IBs to diligently optimise the data presentation in order to ensure the best possible understanding of the IMP characteristics by the investigator and permit an optimal benefit-risk assessment which safeguards those taking part in clinical trials.

**Table 2.** Results of the Online Survey on the Question ‘Do any Parts of the IB Require Improvement (Tick all that Apply)?’ (Multiple Answers Possible).

IB section	Percentage of participants ticking this item (%)
(7) Summary of data and guidance for the investigator	85
(5) Non-clinical studies	76
(6) Effects in humans	56
(2) Summary	44
(4) Physical, chemical, and pharmaceutical properties	18
(3) Introduction	12

## Conclusion

The discussion forum on interpretation and optimisation of the IB for meaningful risk assessment of early clinical trials yielded aspects for future consideration:

- The summary of data and guidance for the investigator are considered as the IB section with the highest need for improvement especially with respect to readability, comprehensibility, timeliness of data, and appropriateness for risk assessment.
- The name and dated signature of at least the sponsor’s scientist responsible for the content on pharmacology and toxicology of the IMP should be given in the IB.
- Changes to the IB should be thoroughly considered whether they constitute substantial amendments, which need regulatory approval.
- All non-clinical pharmacology studies with negative outcomes should be reported in the IB in order to avoid assessment bias.
- The RSI should be depicted as a clearly separate section close to the section 7 of the IB rather than as a subsection thereof.
- The IB should include a section on the amendment change history at the end of the document.
